# Out of the Depths

**Published:** 1888-04-14

**Authors:** N. Grant


### OUT OF THE DEPTHS.

Another gone! gone from our love and care,
Oh God! why giv'st Thou them, our hearth and heart to share,
To take them hence and leave us mad in our despair?

Could'st Thou not find a soul less fondly dear,
Who ne'er on earth a parent's heart might cheer,
To whom Thy world so fair were dark and drear?

Our little son, with life so gladly blest,
Why take him home so soon—so soon to rest?
Oh God! 'tis hard to say "Thy way is best."

Forgive us, Lord, if in our bitter woe
We grudge to Thee the gift Thou did'st bestow;
For every joy it brought to Thee we owe.

That he was ours, our loneness to beguile,
A few short years to glad us with his smile,
We thank Thee, though our heart must break the while.

—*N. Grant.*

